# Supplementary material for: Towards unified AI-driven fracture mechanics: the extended deep energy method (XDEM)
Source: Nat Commun. 2026 Aug 15;17:8492. doi: 10.1038/s41467-026-76748-1 (PMC13484471; doi:10.1038/s41467-026-76748-1)
Supplement: Supplementary file 3 [file 41467_2026_76748_MOESM3_ESM.zip › pic/Results/Stress_intensity_factor/Cross/XDEM/angle_60/crack_u2_displacement.pdf]

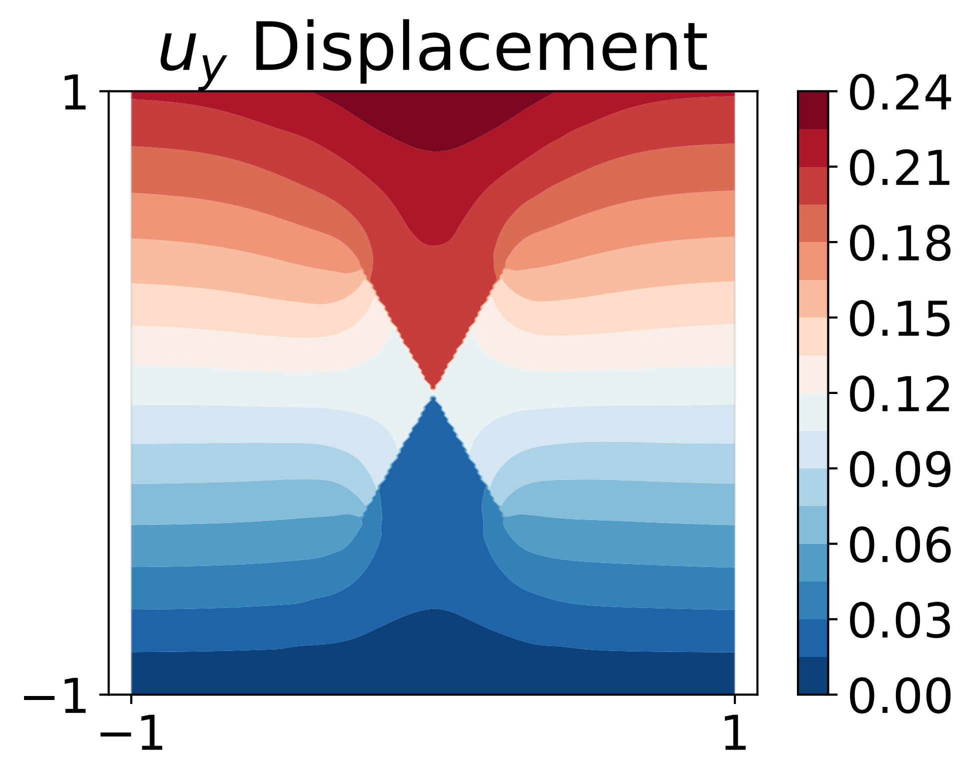
u_y Displacement
1
−1
−1
1
0.24
0.21
0.18
0.15
0.12
0.09
0.06
0.03
0.00
